# Supplementary material for: Astrocytic insulin receptor controls circadian behavior via dopamine signaling in a sexually dimorphic manner
Source: Nat Commun. 2023 Dec 9;14:8175. doi: 10.1038/s41467-023-44039-8 (PMC10710518; doi:10.1038/s41467-023-44039-8)
Supplement: Supplementary file 1 — Supplementary Information [file 41467_2023_44039_MOESM1_ESM.pdf]

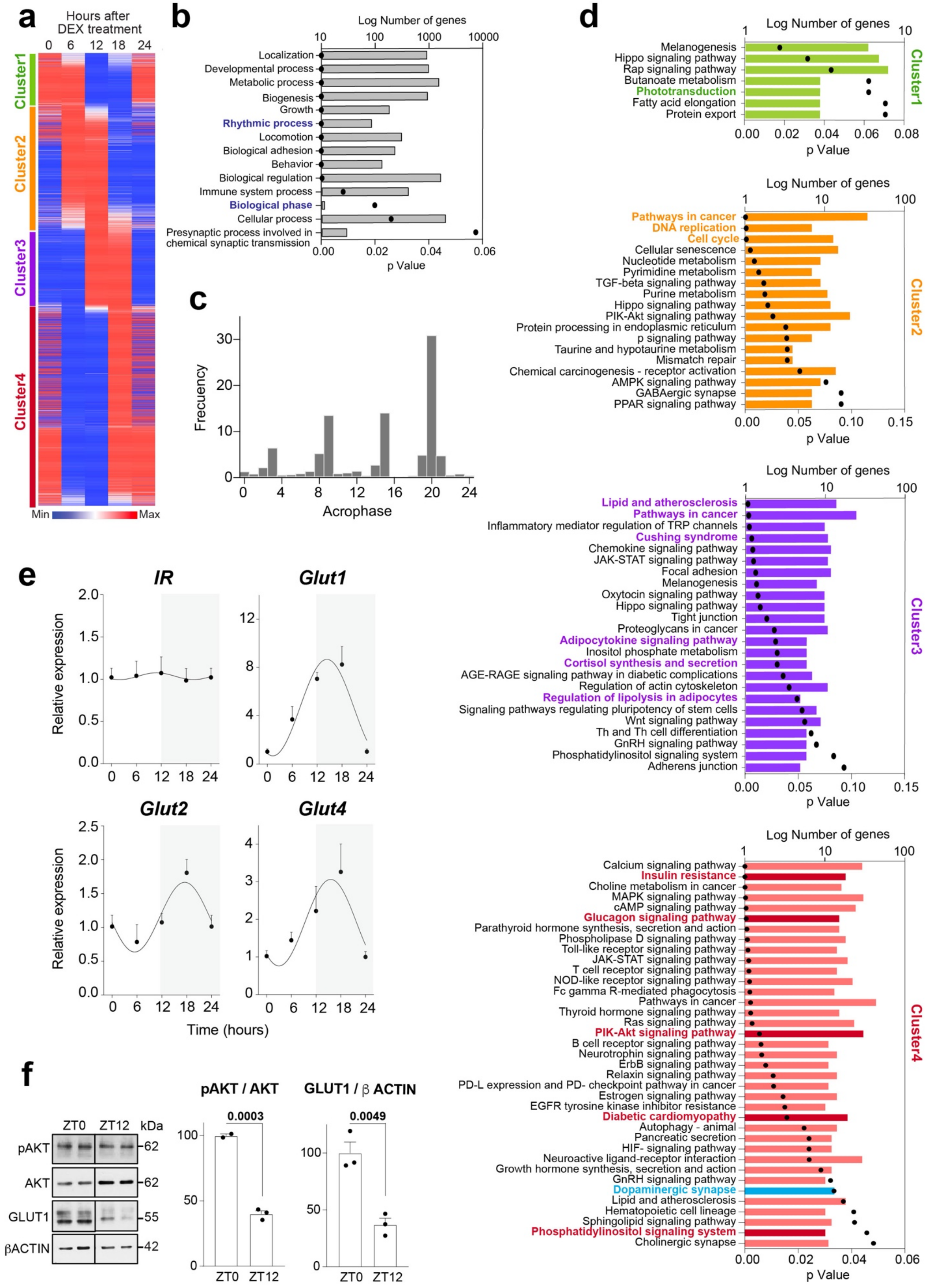

a
Hours after DEX treatment
0 6 12 18 24
Cluster1
Cluster2
Cluster3
Cluster4
Min Max
b
Log Number of genes
10 100 1000 10000
Localization
Developmental process
Metabolic process
Biogenesis
Growth
Rhythmic process
Locomotion
Biological adhesion
Behavior
Biological regulation
Immune system process
Biological phase
Cellular process
Presynaptic process involved in chemical synaptic transmission
0.00 0.02 0.04 0.06
p Value
c
Frecuency
Acrophase
d
Log Number of genes
p Value
Melanogenesis
Hippo signaling pathway
Rap signaling pathway
Butanoate metabolism
Phototransduction
Fatty acid elongation
Protein export
Pathways in cancer
DNA replication
Cell cycle
Cellular senescence
Nucleotide metabolism
Pyrimidine metabolism
TGF-beta signaling pathway
Purine metabolism
Hippo signaling pathway
PIK-Akt signaling pathway
Protein processing in endoplasmic reticulum
p signaling pathway
Taurine and hypotaurine metabolism
Mismatch repair
Chemical carcinogenesis - receptor activation
AMPK signaling pathway
GABAergic synapse
PPAR signaling pathway
Lipid and atherosclerosis
Pathways in cancer
Inflammatory mediator regulation of TRP channels
Cushing syndrome
Chemokine signaling pathway
JAK-STAT signaling pathway
Focal adhesion
Melanogenesis
Oxytocin signaling pathway
Hippo signaling pathway
Tight junction
Proteoglycans in cancer
Adipocytokine signaling pathway
Inositol phosphate metabolism
Cortisol synthesis and secretion
AGE-RAGE signaling pathway in diabetic complications
Regulation of actin cytoskeleton
Regulation of lipolysis in adipocytes
Signaling pathways regulating pluripotency of stem cells
Wnt signaling pathway
Th and Th cell differentiation
GnRH signaling pathway
Phosphatidylinositol signaling system
Adherens junction
Calcium signaling pathway
Insulin resistance
Choline metabolism in cancer
MAPK signaling pathway
cAMP signaling pathway
Glucagon signaling pathway
Parathyroid hormone synthesis, secretion and action
Phospholipase D signaling pathway
Toll-like receptor signaling pathway
JAK-STAT signaling pathway
T cell receptor signaling pathway
NOD-like receptor signaling pathway
Fc gamma R-mediated phagocytosis
Pathways in cancer
Thyroid hormone signaling pathway
Ras signaling pathway
PIK-Akt signaling pathway
B cell receptor signaling pathway
Neurotrophin signaling pathway
ErbB signaling pathway
Relaxin signaling pathway
PD-L expression and PD- checkpoint pathway in cancer
Estrogen signaling pathway
EGFR tyrosine kinase inhibitor resistance
Diabetic cardiomyopathy
Autophagy - animal
Pancreatic secretion
HIF- signaling pathway
Neuroactive ligand-receptor interaction
Growth hormone synthesis, secretion and action
GnRH signaling pathway
Dopaminergic synapse
Lipid and atherosclerosis
Hematopoietic cell lineage
Sphingolipid signaling pathway
Phosphatidylinositol signaling system
Cholinergic synapse
e
IR
Glut1
Glut2
Glut4
Relative expression
Time (hours)
f
ZT0 ZT12 kDa
pAKT 62
AKT 62
GLUT1 55
βACTIN 42
pAKT / AKT
0.0003
GLUT1 / β ACTIN
0.0049
ZT0 ZT12

Supplementary Figure 1. Rhythmic expression of transcripts associated with INS and dopaminergic signaling in synchronous astrocytes. a Heat map of rhythmic astrocyte transcripts. Synchronized astrocytes were harvested at 0.25, 6, 12, 18, and 24 hours after dexamethasone (DEX) treatment. Cluster analysis subdivided the circadian transcripts into four clusters. **b** The biological processes associated with rhythmic transcripts were enriched with terms related to localization, developmental metabolism, and rhythmic processes. The number of genes and p-values for enrichment for each group are indicated. **c** Peak expression phase distribution of prevalent rhythmic transcripts in primary synchronous astrocytes. **d** KEGG pathways enrichment of rhythmic transcripts in the four clusters, showing that transcripts of the fourth cluster associate with INS signaling and dopaminergic synapses. The number of genes and p-values for enrichment for each group are indicated. **e** Analysis of *IR, Glut1, Glut2 and Glut4* levels in the hypothalamus of control animals in regular LD cycles by quantitative RT-PCR. Grey areas indicate the dark phase. The ZT24 time point is the ZT0 time point, shown again. Graphs show the mean ± s.e.m. of the cosine-fitted curves from three experiments performed in duplicate. **f** Protein levels of GLUT1, pAKT and AKT in the hypothalamus of control animals in regular LD cycles at ZT0 (n = 2 mice for pAKT and AKT; n = 3 mice for GLUT1) and ZT12 (n = 3 mice) are presented. Representative images for all proteins are shown; all the bands for each protein are derived from the same gel, although they were spliced for clarity. Graphs show the mean ± s.e.m. Two-tailed, unpaired t-test. Source data are provided as a Source Date file.

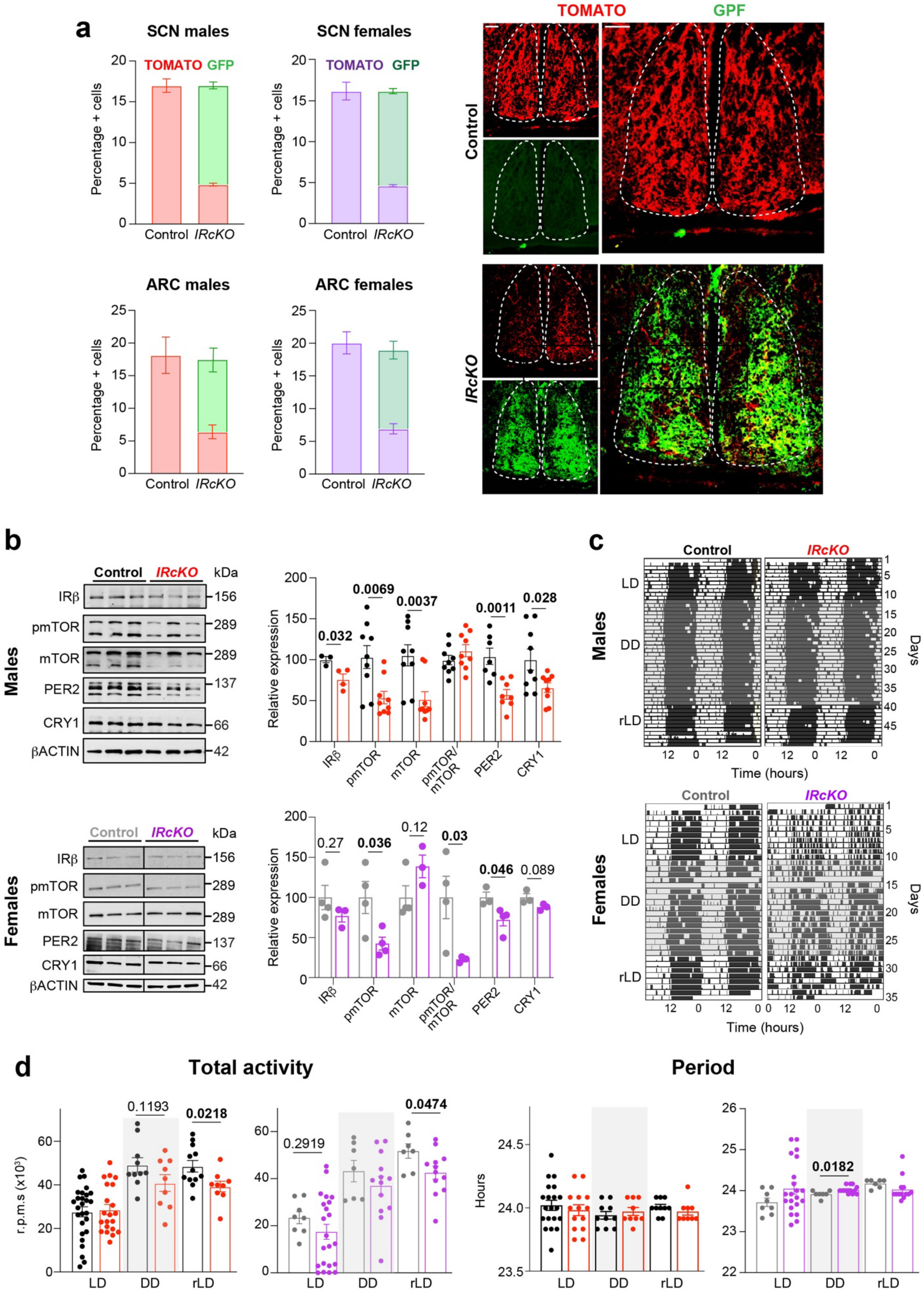

a
SCN males
SCN females
TOMATO GFP
Percentage + cells
Control IRcKO
ARC males
ARC females
TOMATO
GPF
Control
IRcKO
b
Males
Females
Control IRcKO kDa
IRβ
pmTOR
mTOR
PER2
CRY1
βACTIN
156
289
137
66
42
Relative expression
0.032
0.0069
0.0037
0.0011
0.028
0.27
0.036
0.12
0.03
0.046
0.089
pmTOR/ mTOR
c
LD
DD
rLD
Days
Time (hours)
d
Total activity
Period
r.p.m.s (x10³)
Hours
0.1193
0.0218
0.2919
0.0474
0.0182

Supplementary Figure 2. Cre-mediated recombination in the SCN and circadian locomotor activity of control and *IRcKO*. a Quantification of the percentage of TOMATO- or GFP-positive cells in the SCN and ARC in control or *IRcKO-tdTomato/eGFP* males (SCN: n = 6 per group; ARC: n = 6 controls and n = 7 mutants) and females (SCN: n = 10 control and n = 8 mutants; ARC: n = 8 control and n = 6 mutants). Representative micrographs show TOMATO and GFP expression in the SCN of control and *IRcKO-tdTomato/eGFP* mice. Scale bars, 50 µm. **b** Representative images and quantification of hypothalamic IRβ, phospho-mTOR, mTOR, PER2, and CRY1 western blots in *IRcKO* males (upper panel) and females (lower panel) compared to control mice, 2 months after TM treatment. IRβ: n = 3-4 males and n = 4-3 females in control-mutant pairs; pmTOR and mTOR: n = 9 males and n = 4 females per group; CRY1: n = 9 males and n = 3 females per group; PER2: n = 7-8 males and n = 3-4 females in control-mutant pairs. All the bands of each protein are derived from the same gel, however, for the females they were spliced for clarity. Two-tailed, unpaired t-test. **c** Representative actograms of control and *IRcKO* mice under LD, DD and rLD cycles. **d** Total activity and periodicity of control and *IRcKO* mice in LD, DD, and rLD cycles. Total activity: males: n = 27-21 (LD), n = 10-9 (DD), and n = 12-9 (rLD) animals in control-mutant pairs; females: n = 8-22 (LD), n = 7-13 (DD), and n = 7-12 (rLD) animals in control-mutant pairs. Periodicity: males: n = 20-14 (LD) and n = 10-9 (DD and rLD) animals in control-mutant pairs; females: n = 8-21 (LD) and n = 7-13 (DD and rLD) animals in control-mutant pairs. Two-tailed, unpaired t-test. Data are represented as mean ± s.e.m. Source data are provided as a Source Date file.

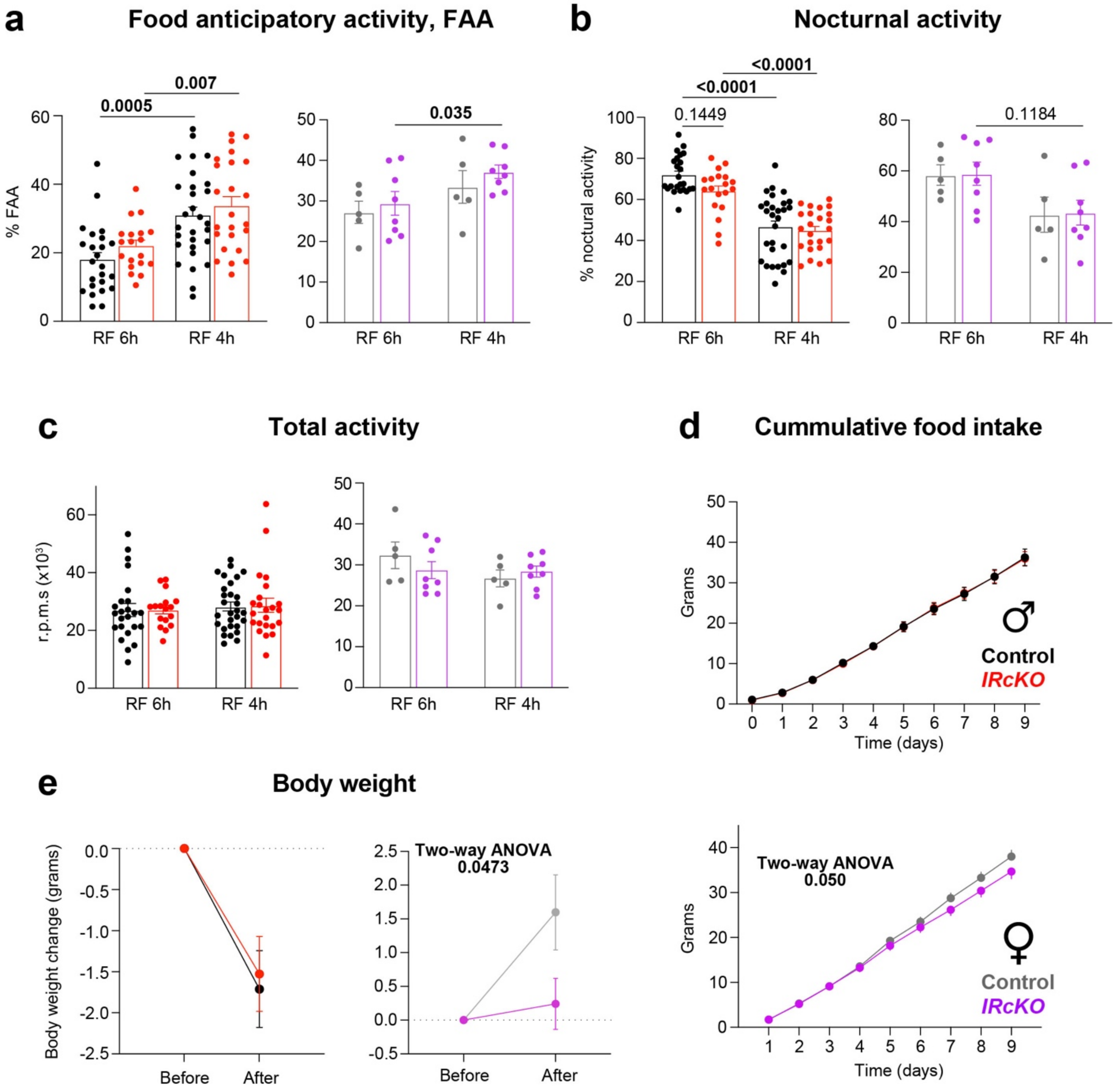

Supplementary Figure 3. Activity, food intake, and body weight change of control and *IRcKO* animals subjected to the RF paradigm. Percentage of the FAA (**a**), nocturnal activity (**b**), and total activity (**c**) in control and *IRcKO* mice. For males, FAA and nocturnal activity, n = 24-19 (RF6h), n = 29-24 (RF4h) in control-mutant pairs; and total activity: n = 24-17 (RF6h), n = 29-23 (RF4h) in control-mutant pairs. For females, n = 5-8 in control-mutant pairs. Cumulative food intake (**d**) and body weight change (**e**) of control and *IRcKO* mice. Cumulative food intake: males, n = 24-19 and females, n = 5-9 in control-mutant pairs. Body weight change: males, n = 19-24 and females n = 5-8 in control-mutant pairs. Data are represented as mean ± s.e.m. Two-way ANOVA or two-tailed, unpaired t-test. Source data are provided as a Source Date file.

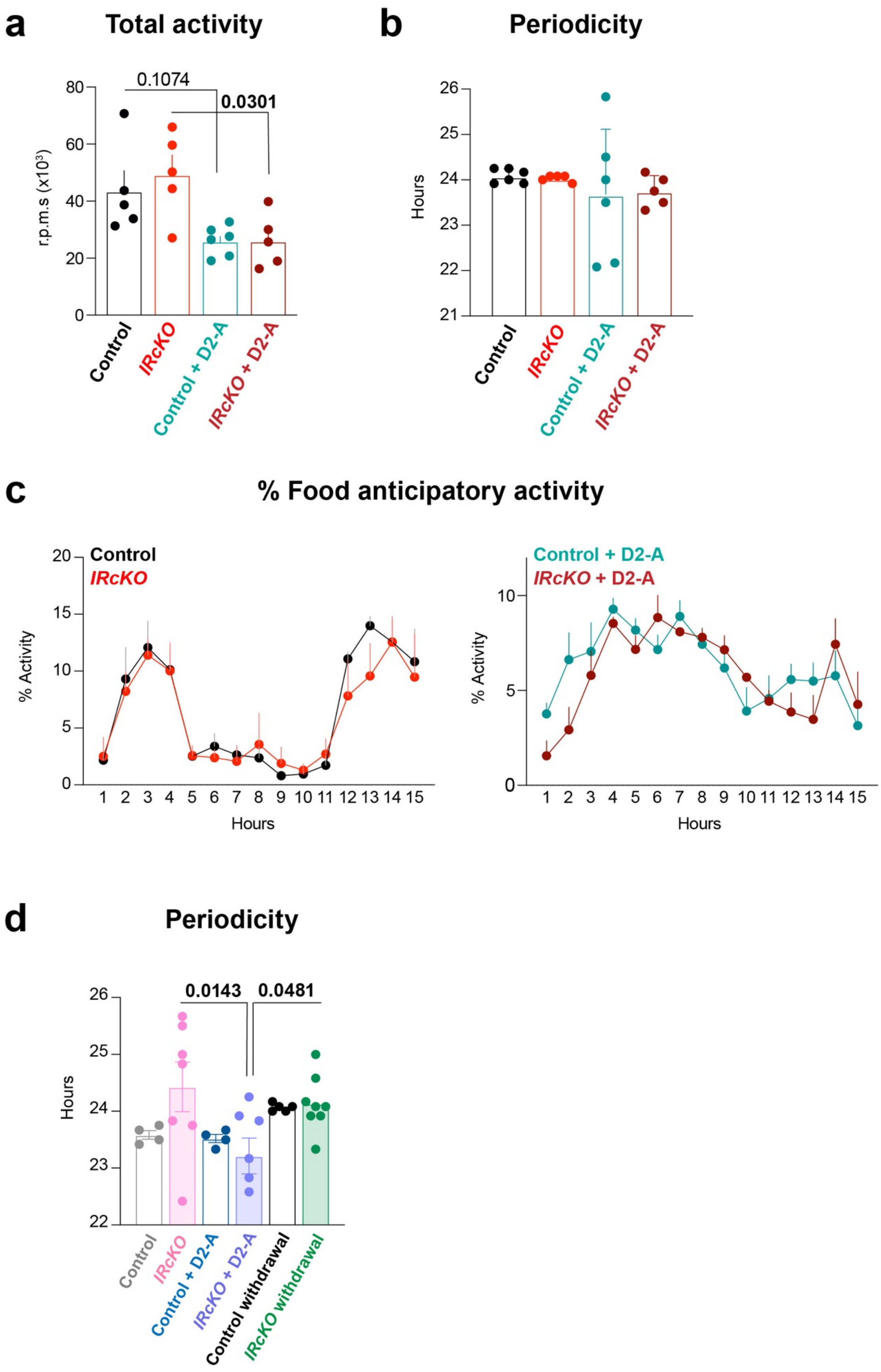
a
Total activity
b
Periodicity
0.1074
0.0301
r.p.m.s (x10³)
Hours
Control
IRcKO
Control + D2-A
IRcKO + D2-A
c
% Food anticipatory activity
Control
IRcKO
Control + D2-A
IRcKO + D2-A
% Activity
Hours
d
Periodicity
0.0143
0.0481
Hours
Control
IRcKO
Control + D2-A
IRcKO + D2-A
Control withdrawal
IRcKO withdrawal

Supplementary Figure 4. **Locomotor activity and periodicity of *IRcKO* mice treated with a DRD2 agonist.** (**a**) Total activity and (**b**) periodicity of male control (n = 5 and 6) and *IRcKO* (n = 5) mice treated with the DRD2 agonist quinpirole (1mg/kg per day) or vehicle for 5 days. One way-ANOVA. **c** Percentage of activity of control and *IRcKO* males during the restricted feeding paradigm and after the treatment with the DRD2 agonist quinpirole (1mg/kg per day) (n = 6) or vehicle at ZT12 (n = 7 and 5). **d** Periodicity of control and *IRcKO* female mice treated with the DRD2 agonist quinpirole (1mg/kg per day) or vehicle for 5 days (n = 4 and 7) and after the drug withdrawal (7 days) (n = 5 and 8). Two-way ANOVA. Data are represented as mean ± s.e.m. Source data are provided as a Source Date file.

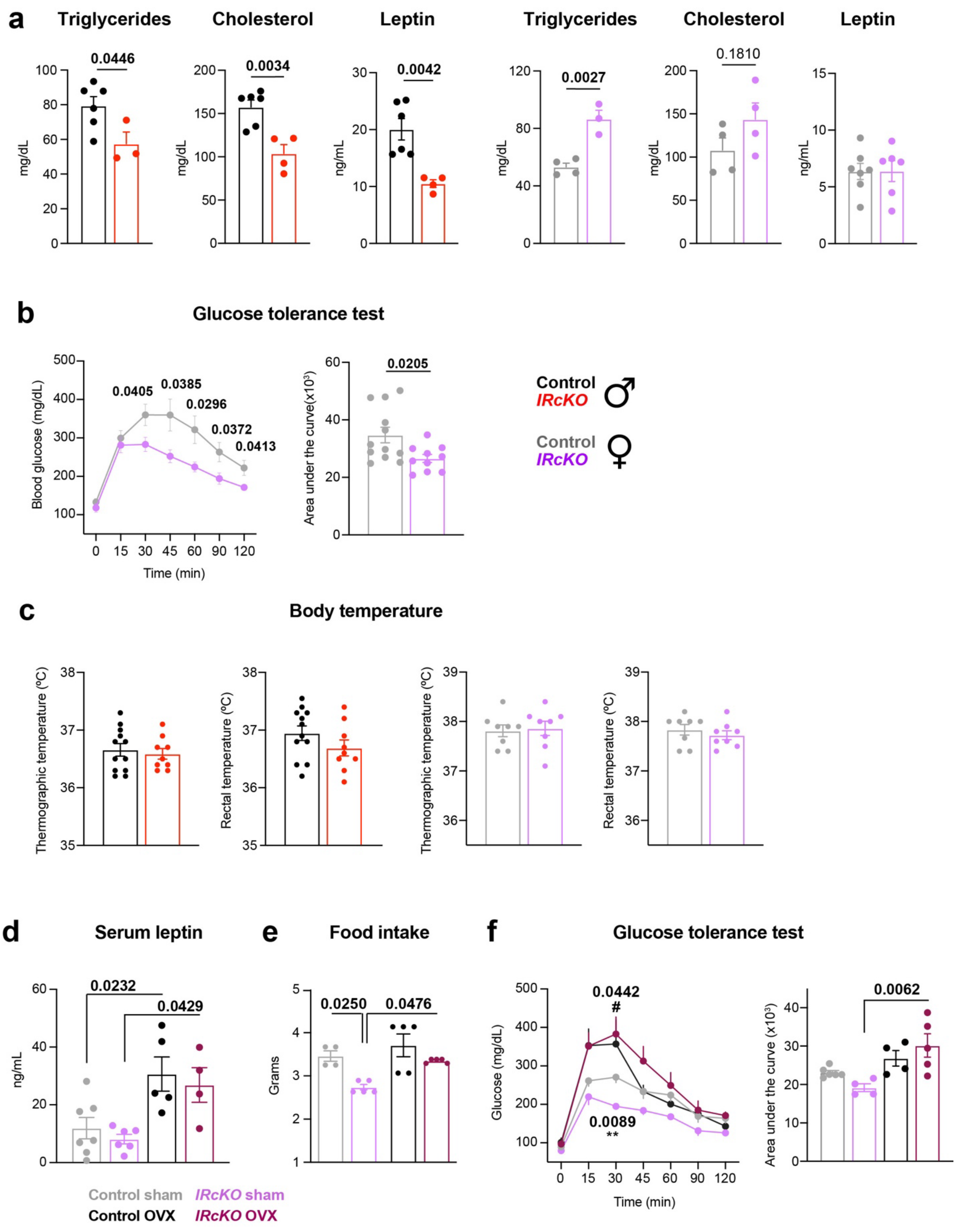
a
Triglycerides
Cholesterol
Leptin
Triglycerides
Cholesterol
Leptin
0.0446
0.0034
0.0042
0.0027
0.1810
mg/dL
ng/mL
b
Glucose tolerance test
Blood glucose (mg/dL)
Time (min)
0.0405
0.0385
0.0296
0.0372
0.0413
Area under the curve(x10³)
0.0205
Control
IRcKO
Control
IRcKO
c
Body temperature
Thermographic temperature (ºC)
Rectal temperature (ºC)
Thermographic temperature (ºC)
Rectal temperature (ºC)
d
Serum leptin
ng/mL
0.0232
0.0429
e
Food intake
Grams
0.0250
0.0476
f
Glucose tolerance test
Glucose (mg/dL)
Time (min)
0.0442
#
0.0089
**
Area under the curve (x10³)
0.0062
Control sham
IRcKO sham
Control OVX
IRcKO OVX

Supplementary Figure 5. Serum triglycerides, cholesterol and leptin, glucose tolerance test, body temperature and food intake in *IRcKO* mice and OVX females. **a** Serum triglycerides, cholesterol, and leptin levels in control (n = 4-6) and *IRcKO* (n = 3-6) mice. Two-tailed, unpaired t-test. **b** Glucose tolerance test in control (n =12) and *IRcKO* (n = 10) female mice 13 weeks after TM treatment, performed at ZT10. Two-tailed, unpaired t-test. **c** BAT and rectal temperature of control (n = 12 males and n = 8 females) and *IRcKO* (n = 9 males and n = 8 females) mice 11 weeks after TM treatment. **d** Serum leptin levels in control (n = 5 and 7) and *IRcKO* (n = 4 and 6) females 13 weeks after sham surgery or OVX. Two-way ANOVA. **e** Food intake of sham and OVX control (n = 4 and 5) and *IRcKOs* (n = 5), 6 weeks after surgical procedures. Two-way ANOVA. **f** Glucose tolerance test of sham and OVX control (n = 6 and 4) and *IRcKO* (n = 4 and 5) females 12 weeks after OVX or sham surgery, performed at ZT10. Two-way ANOVA (** p = 0.0089 vs. sham controls; # p = 0.0442 sham *IRcKO* mice). Data are represented as mean ± s.e.m. Source data are provided as a Source Date file.

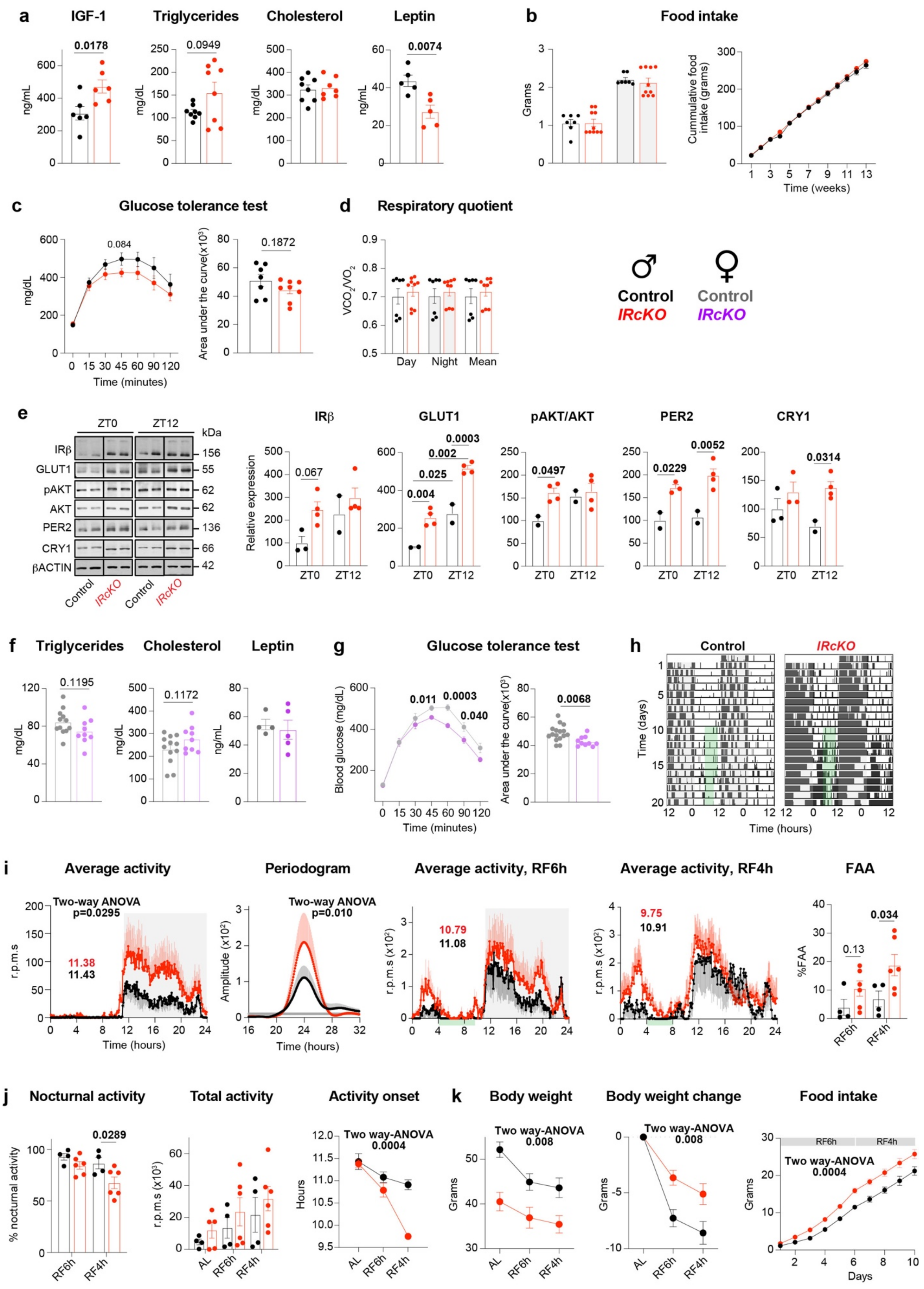

a
IGF-1
Triglycerides
Cholesterol
Leptin
0.0178
0.0949
0.0074
ng/mL
mg/dL
b
Food intake
Grams
Cummulative food intake (grams)
Time (weeks)
c
Glucose tolerance test
0.084
mg/dL
Time (minutes)
Area under the curve(x10³)
0.1872
d
Respiratory quotient
VCO2/VO2
Day
Night
Mean
♂
♀
Control
IRcKO
Control
IRcKO
e
ZT0
ZT12
kDa
IRβ
GLUT1
pAKT
AKT
PER2
CRY1
βACTIN
156
55
62
62
136
66
42
Control
IRcKO
Control
IRcKO
IRβ
GLUT1
pAKT/AKT
PER2
CRY1
Relative expression
0.067
0.0003
0.002
0.025
0.004
0.0497
0.0229
0.0052
0.0314
f
Triglycerides
Cholesterol
Leptin
0.1195
0.1172
mg/dL
ng/mL
g
Glucose tolerance test
0.011
0.0003
0.040
Blood glucose (mg/dL)
Time (minutes)
Area under the curve(x10³)
0.0068
h
Control
IRcKO
Time (days)
Time (hours)
i
Average activity
Periodogram
Average activity, RF6h
Average activity, RF4h
FAA
Two-way ANOVA p=0.0295
Two-way ANOVA p=0.010
11.38
11.43
10.79
11.08
9.75
10.91
r.p.m.s
Amplitude (x10²)
r.p.m.s (x10²)
Time (hours)
%FAA
0.13
0.034
RF6h
RF4h
j
Nocturnal activity
Total activity
Activity onset
% nocturnal activity
0.0289
r.p.m.s (x10³)
Two way-ANOVA 0.0004
Hours
AL
RF6h
RF4h
k
Body weight
Body weight change
Food intake
Two way-ANOVA 0.008
Two way-ANOVA 0.008
Two way-ANOVA 0.0004
Grams
RF6h
RF4h
Days

Supplementary Figure 6. Astrocytic IR differentially regulates energy balance in DIO. a Serum IGF-1 (n = 6), triglycerides (n = 7 and 8), cholesterol (n = 8 and 7), and leptin (n = 5) in DIO-challenged control and *IRcKO* males at 20 weeks on HFD. Two-tailed, unpaired t-test. **b** Daily food intake (n = 7 and 10) and cumulative food intake (n = 7 and 11) of DIO-challenged control and *IRcKO* males. **c** Glucose tolerance test in DIO-challenged control (n = 7) and *IRcKO* (n = 8) male mice at 11 weeks on HFD, performed at ZT10. Two-tailed, unpaired t-test. **d** Respiratory quotient of DIO-challenged control (n = 7) and *IRcKO* (n = 9) male mice. **e** Representative images and quantifications of hypothalamic IRβ, GLUT1, phospho-AKT, AKT, PER2, and CRY1 western blots in *IRcKO* compared to control mice at ZT0 and ZT12 (n = 2-4). Two-way ANOVA. **f** Serum triglycerides (n = 12 and 9), cholesterol (n = 12 and 9), and leptin (n = 4 and 5) in DIO-challenged control and *IRcKO* females at 26 weeks on HFD. Two-tailed, unpaired t-test. **g** Glucose tolerance test in DIO-challenged control (n = 16) and *IRcKO* (n = 10) female mice at 11 weeks on HFD, performed at ZT10. Two-tailed, unpaired t-test. **h** Representative actograms depicting the circadian behavior of control and *IRcKO* mice *ad libitum* and during the RF paradigm. **i** Average waveforms of DIO-challenged control and *IRcKO* mice *ad libitum* (n = 4 and 5) and under RF from ZT 4-10 (RF 6h) and ZT 4-8 (RF 4h) (n = 4 and 6). Periodogram of DIO-challenged control and *IRcKO* mice *ad libitum* (n = 4 and 5). Grey areas indicate the nighttime and green areas the feeding time. The onset of activity is indicated in the graphs. Percentage of the FAA of DIO-challenged control (n = 4) and *IRcKO* (n = 6) mice under the RF paradigm. Two way-ANOVA or two-tailed, unpaired t-test. **j** Percentage of nocturnal activity, total activity, and activity onset of DIO-challenged control (n = 4) and *IRcKO* (n = 6) mice under the RF paradigm. Two way-ANOVA. **k** Body weights and cumulative food intake of DIO-challenged control (n = 4) and *IRcKO* (n = 6) mice under the RF paradigm. Two way-ANOVA. Data are represented as mean ± s.e.m. Source data are provided as a Source Date file.

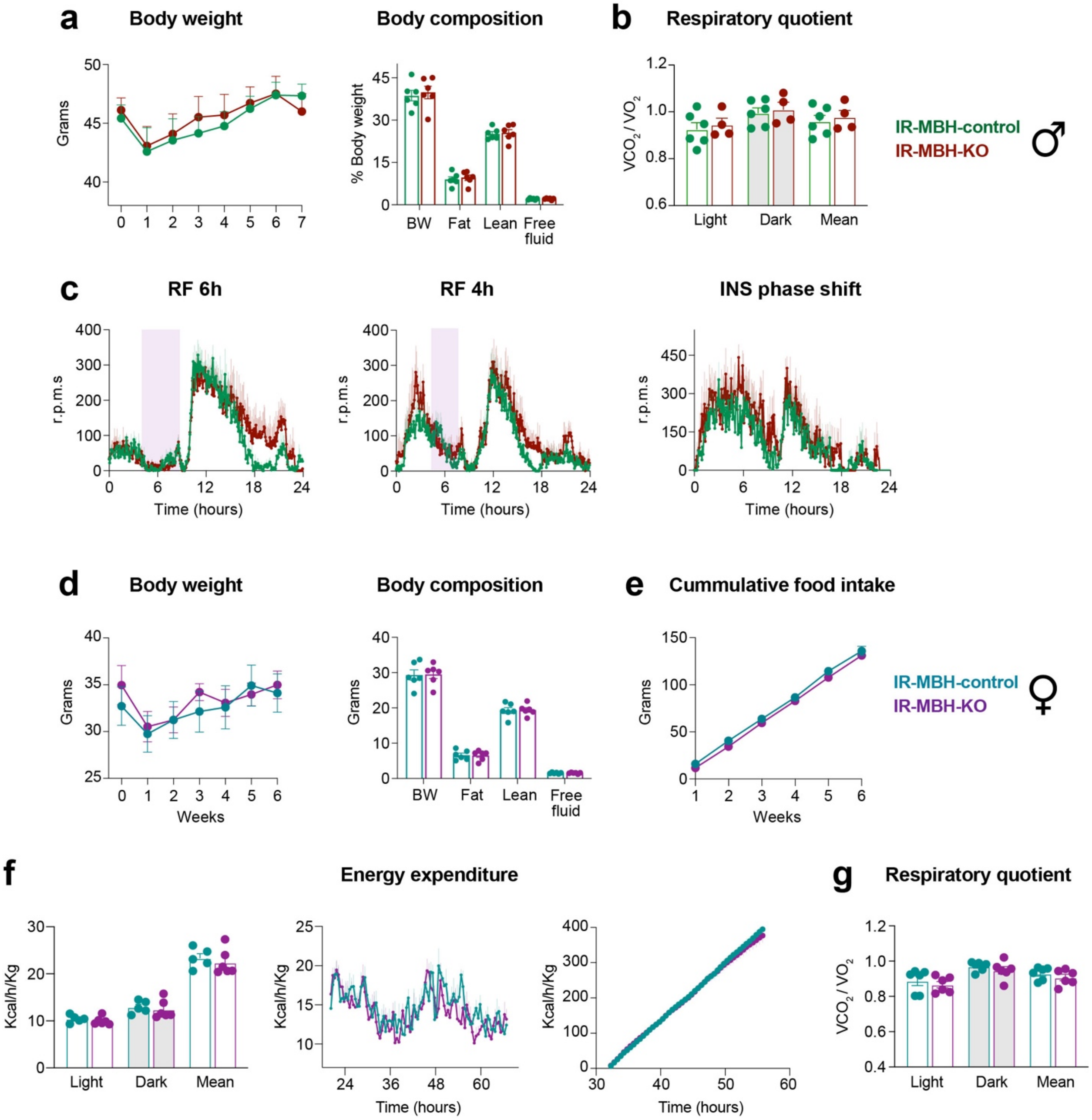

Supplementary Figure 7. Metabolic phenotype of mice with astrocytic IR ablation in the MBH. Age-dependent changes in body weight and composition in male (**a**) and female (**d**) IR-MBH-control or IR-MBH-KO animals (n = 6 mice). **b** Respiratory quotient during the light and dark phase in male control (n = 6 mice) and mutants (n = 4 mice). **c** Average waveforms of control and mutant mice under food restriction (RF) from ZT 4-10 (RF 6h, n = 5 and 6 mice), ZT 4-8 (RF 4h, n = 6 and 4 mice), and after the INS and glucose bolus administration (n = 6 mice). Purple areas in the graphs indicate the feeding time. **e** Cumulative food intake in females IR-MBH-control or IR-MBH-KO mice (n = 6 animals). **f** Hourly plots show hourly energy expenditure, and bar plots show total energy expenditure in female control (n = 5 mice) and mutants (n = 6 mice). **g** Respiratory quotient of IR-MBH-control or IR-MBH-KO females (n = 6 mice). Source data are provided as a Source Date file.

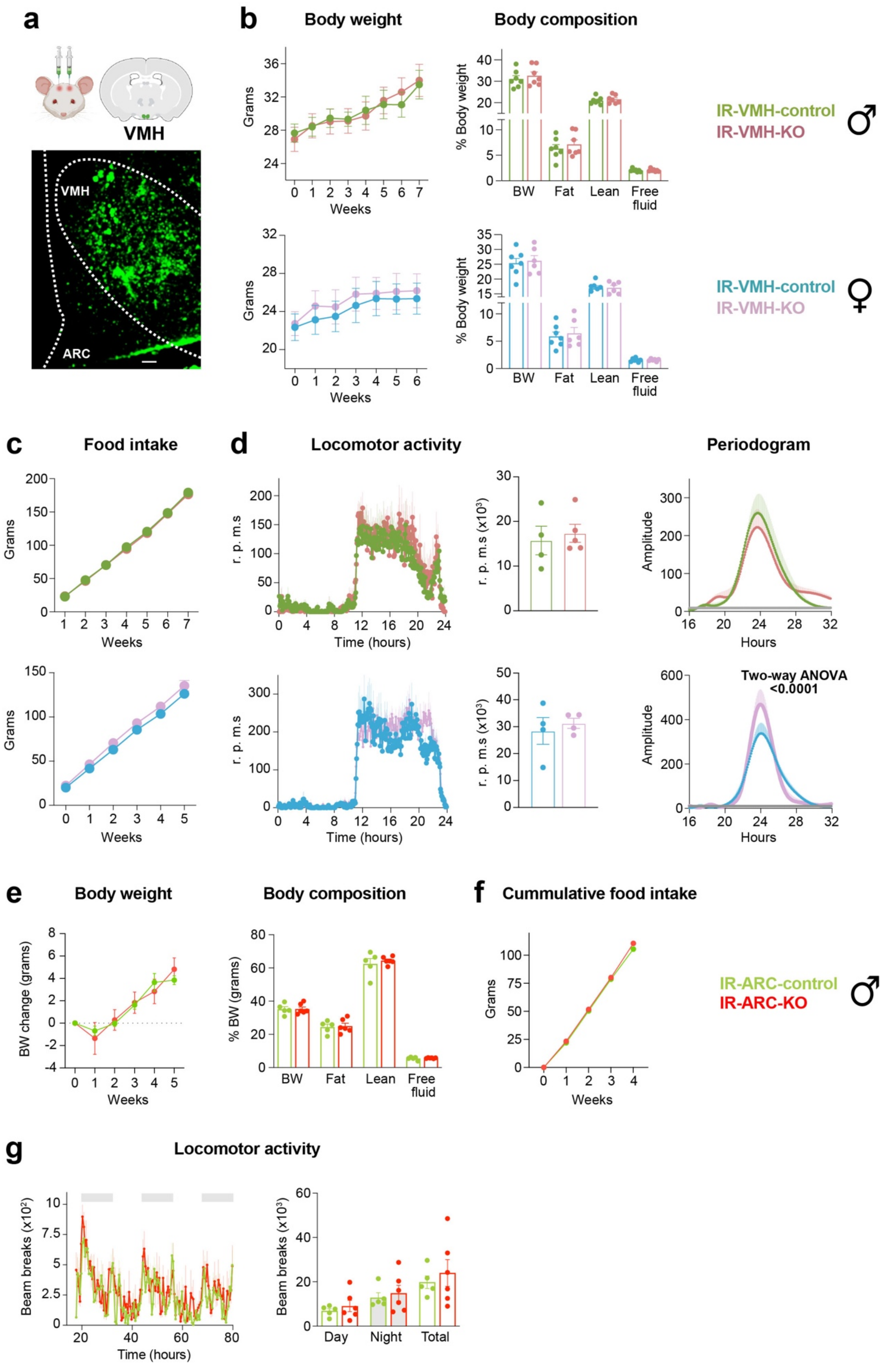
a
VMH
ARC
b
Body weight
Body composition
Grams
Weeks
% Body weight
BW
Fat
Lean
Free fluid
IR-VMH-control
IR-VMH-KO
c
Food intake
d
Locomotor activity
Periodogram
r. p. m.s
Time (hours)
r. p. m.s (x10³)
Amplitude
Hours
Two-way ANOVA
<0.0001
e
Body weight
BW change (grams)
Body composition
% BW (grams)
f
Cummulative food intake
IR-ARC-control
IR-ARC-KO
g
Locomotor activity
Beam breaks (x10²)
Beam breaks (x10³)
Day
Night
Total

Supplementary Figure 8. Metabolic phenotype of mice with astrocytic IR ablation in the VMH and ARC. a VMH showing AAV-GFAP-GFP infection in $IR^{fl/fl}$ mice. Scale bar: 100µm. **b** Body weight and composition in IR-VMH-control and IR-VMH-KO mice (n = 7 males; n = 7 and 6 females). Cumulative food intake (n = 7 males; n = 7 and 6 females) (**c**), locomotor activity and periodogram (**d**) in controls (n = 4) and mutants (n = 5 males and n = 4 females). **e** Body weight and composition in AAV-ARC-controls (n = 5 mice) and AAV-ARC-KO (n = 6 mice) males. Cumulative food intake (**f**) and locomotor activity (**g**) in control and AAV-ARC-KO males (n = 5 control and n = 6 mutant mice). Data are represented as mean ± s.e.m. Mouse head and coronal mouse brain (a) were created with Biorender.com. Source data are provided as a Source Date file.
