# Supplementary material for: Astrocytic insulin receptor controls circadian behavior via dopamine signaling in a sexually dimorphic manner
Source: Nat Commun. 2023 Dec 9;14:8175. doi: 10.1038/s41467-023-44039-8 (PMC10710518; doi:10.1038/s41467-023-44039-8)
Supplement: Supplementary file 3 — Description of Additional Supplementary Files [file 41467_2023_44039_MOESM3_ESM.docx]

**Description of Additional Supplementary Files**

**Supplementary Data 1:** Rhythmic Transcripts in DEX-Synchronized Astrocytes

**Supplementary Data 2:** List of astrocyte circadian transcripts related to rhythmic processes

**Supplementary Data 3:** List of astrocyte circadian transcripts related to INS signalling

**Supplementary Data 4:** List of astrocyte circadian transcripts related to dopaminergic synapse

**Supplementary Data 5:** Primer sequences used for real-time quantitative reverse transcriptase polymerase chain reaction (qRT-PCR).
