# Supplementary material for: Astrocytic insulin receptor controls circadian behavior via dopamine signaling in a sexually dimorphic manner
Source: Nat Commun. 2023 Dec 9;14:8175. doi: 10.1038/s41467-023-44039-8 (PMC10710518; doi:10.1038/s41467-023-44039-8)
Supplement: Supplementary file 9 — Reporting Summary [file 41467_2023_44039_MOESM9_ESM.pdf]

nature portfolio

Corresponding author(s): Olga Barca Mayo and Miguel López

Last updated by author(s): Oct 30, 2023

Reporting Summary

Nature Portfolio wishes to improve the reproducibility of the work that we publish. This form provides structure for consistency and transparency in reporting. For further information on Nature Portfolio policies, see our Editorial Policies and the Editorial Policy Checklist.

Statistics

For all statistical analyses, confirm that the following items are present in the figure legend, table legend, main text, or Methods section.

n/a	Confirmed	
☐	☒	The exact sample size (n) for each experimental group/condition, given as a discrete number and unit of measurement
☐	☒	A statement on whether measurements were taken from distinct samples or whether the same sample was measured repeatedly
☐	☒	The statistical test(s) used AND whether they are one- or two-sided<br>*Only common tests should be described solely by name; describe more complex techniques in the Methods section.*
☐	☒	A description of all covariates tested
☐	☒	A description of any assumptions or corrections, such as tests of normality and adjustment for multiple comparisons
☐	☒	A full description of the statistical parameters including central tendency (e.g. means) or other basic estimates (e.g. regression coefficient) AND variation (e.g. standard deviation) or associated estimates of uncertainty (e.g. confidence intervals)
☐	☒	For null hypothesis testing, the test statistic (e.g. F, t, r) with confidence intervals, effect sizes, degrees of freedom and P value noted<br>*Give P values as exact values whenever suitable.*
☒	☐	For Bayesian analysis, information on the choice of priors and Markov chain Monte Carlo settings
☒	☐	For hierarchical and complex designs, identification of the appropriate level for tests and full reporting of outcomes
☒	☐	Estimates of effect sizes (e.g. Cohen's d, Pearson's r), indicating how they were calculated

Our web collection on statistics for biologists contains articles on many of the points above.

Software and code

Policy information about availability of computer code

Data collection	Data were acquired with commercially available softwares indicated in the Methods section: Wheel Manager software (SOF-860; Med Associates, Inc) and LabMaster (TSE Systems).
Data analysis	The data were analyzed using a combination of commercially available software, including Microsoft Excel, Graphpad Prism, and FLIR Tools, as well as open-source software such as ImageJ, Actogram J (Fiji 5 Plugin), BIO_CYCLE cosine analysis (http://circadiomics.igb.uci.edu/), and DAVID Bioinformatics Resources (https://david.ncifcrf.gov/).

For manuscripts utilizing custom algorithms or software that are central to the research but not yet described in published literature, software must be made available to editors and reviewers. We strongly encourage code deposition in a community repository (e.g. GitHub). See the Nature Portfolio guidelines for submitting code & software for further information.

Data

Policy information about availability of data

All manuscripts must include a data availability statement. This statement should provide the following information, where applicable:

- Accession codes, unique identifiers, or web links for publicly available datasets
- A description of any restrictions on data availability
- For clinical datasets or third party data, please ensure that the statement adheres to our policy

All data generated in this study are provided in the Source Data file. Source data are provided with this paper. The microarray data used in this study are available in the NCBI Gene Expression Omnibus database under accession code GSE39272.

Human research participants

Policy information about studies involving human research participants and Sex and Gender in Research.

Reporting on sex and gender	N/A
Population characteristics	N/A
Recruitment	N/A
Ethics oversight	N/A

Note that full information on the approval of the study protocol must also be provided in the manuscript.

Field-specific reporting

Please select the one below that is the best fit for your research. If you are not sure, read the appropriate sections before making your selection.

[x] Life sciences [ ] Behavioural & social sciences [ ] Ecological, evolutionary & environmental sciences

For a reference copy of the document with all sections, see nature.com/documents/nr-reporting-summary-flat.pdf

Life sciences study design

All studies must disclose on these points even when the disclosure is negative.

Sample size	For animal experiments, sample size was chosen based on similar previous studies of our group and based on literature documentation of similar well characterized experiments. We try to use the fewest number of mice to achieve statistical significance. The sample size and the power to detect the differences between experimental groups are provided in figure legends and in the Statistical Analysis section in Methods.<br>Luengo-Mateos, M. et al. Hypothalamic astrocytic-BMAL1 regulates energy homeostasis in a sex-dependent manner. Cell Reports 42,8 (2023)<br>Barca-Mayo, O. et al. Astrocyte deletion of Bmal1 alters daily locomotor activity and cognitive functions via GABA signalling. Nat. Commun. 8, (2017).<br>García-Cáceres, C. et al. Astrocytic Insulin Signaling Couples Brain Glucose Uptake with Nutrient Availability. Cell 166, 867–880 (2016).<br>Milbank, E., Martinez, M. C. & Andriantsitohaina, R. Extracellular vesicles: pharmacological modulators of the peripheral and central signals governing obesity. Pharmacol. Ther. 157, 65–83 (2016).<br>López, M. et al. Hypothalamic Fatty Acid Metabolism Mediates the Orexigenic Action of Ghrelin. Cell Metab. 7, 389–399 (2008); Heras, V. et al. Central ceramide signaling mediates obesity-induced precocious puberty. Cell Metab. 32, 951-966 e958 (2020).
Data exclusions	Samples or animals were excluded from the data analysis with pre-established criteria, if they deviated more than 2 s.d. from the group mean. Criteria were pre-established.
Replication	Animal experiments were performed with sufficient sample sizes as stated above. Every experiment was repeated at least twice and all attempts to replicate experiments were successful.
Randomization	Before starting an experiment, all mice groups were made with set of animals of the same sex, age and similar body weight. See Statistical analysis section. The distribution of the animals with the same genotype to receive the treatments was randomized.
Blinding	For practical reasons, the investigators were not blinded to in vivo treatments, since we need to know which animals are injected with each treatment. After collecting the samples, the investigators were blinded to group allocation during data collection and analysis

Reporting for specific materials, systems and methods

We require information from authors about some types of materials, experimental systems and methods used in many studies. Here, indicate whether each material, system or method listed is relevant to your study. If you are not sure if a list item applies to your research, read the appropriate section before selecting a response.

Materials & experimental systems

n/a	Involved in the study
☐	☒ Antibodies
☒	☐ Eukaryotic cell lines
☒	☐ Palaeontology and archaeology
☐	☒ Animals and other organisms
☒	☐ Clinical data
☒	☐ Dual use research of concern

Methods

n/a	Involved in the study
☒	☐ ChIP-seq
☒	☐ Flow cytometry
☒	☐ MRI-based neuroimaging

Antibodies

Antibodies used

The following antibodies were used:
PER2 (Invitrogen PA5-89045) (dilution 1:1000)
CRY1 (Invitrogen, PA5-89349) (dilution 1:1000)
IRb (CT-3) (Santa Cruz Biotechnology, sc-57342) (dilution 1:500)
UCP1 (Abcam, ab10983) (dilution 1:1000)
phospho-mTOR (Cell Signaling, 2971-S) (dilution 1:1000)
mTOR (Cell Signaling, 2972-S) (dilution 1:1000)
phospho-AKT (Cell Signaling, 9271) (dilution 1:1000)
AKT (Cell Signaling, 9272) (dilution 1:1000)
GLUT1 (Invitrogen, PA1-46152)
mouse anti-rabbit IgG-HRP (Santa Cruz Biotechnology, sc-2357) (dilution 1:5000)
m-IgG1 BP-HRP: (Santa Cruz Biotechnology, sc-525408) (dilution 1:5000)

All primary antibodies used in this report were obtained from commercial sources.

Validation

The validation information can be found online for antibodies from:
Cell Signaling:
https://www.cellsignal.com/about-us/cst-antibody-validation-principles
phospho-mTOR: https://www.cellsignal.com/products/primary-antibodies/phospho-mtor-ser2448-antibody/2971
mTOR: https://www.cellsignal.com/products/primary-antibodies/mtor-antibody/2972
phospho-AKT: https://www.cellsignal.com/products/primary-antibodies/phospho-akt-ser473-antibody/9271
AKT: https://www.cellsignal.com/products/primary-antibodies/akt-antibody/9272

Abcam:
https://www.abcam.com/primary-antibodies/a-guide-to-antibody-validation
https://www.abcam.com/products/primary-antibodies/ucp1-antibody-ab10983.html

Thermo Fisher:
PER2: https://www.thermofisher.com/antibody/product/PER2-Antibody-Polyclonal/PA5-89045
CRY1: https://www.thermofisher.com/antibody/product/CRY1-Antibody-Polyclonal/PA5-89349
GLUT1: https://www.thermofisher.com/antibody/product/GLUT1-Antibody-Polyclonal/PA1-46152

Santa Cruz Biotechnology
IRbeta: https://www.scbt.com/p/insulin-rbeta-antibody-ct-3
mouse anti-rabbit IgG-HRP: https://datasheets.scbt.com/sc-2357.pdf
m-IgG1 BP-HRP: https://datasheets.scbt.com/sc-525408.pdf

Animals and other research organisms

Policy information about studies involving animals; ARRIVE guidelines recommended for reporting animal research, and Sex and Gender in Research

Laboratory animals

Female and male postnatal day 1–3 Sprague Dawley Rats were housed with their dams in a 12h light–dark cycle in a temperature and

	humidity-controlled room. Water and food (Teklad-7913, Envigo) were provided ad libitum for the dam. Mice were housed with ad libitum access to food and water and kept on a 12-hour (8 A.M. to 8 P.M.) light-dark cycle in a temperature- and humidity-controlled room at the animal facility of the University of Santiago de Compostela.<br><br>Mice having the sequence of the IR gene flanked by loxP sites (IRf/f) (generated by Ronald Kahn, Joslin Diabetes Center) were crossed with the GlastCreERT2 mouse line and with the Rosa26 ACTB-tdTomato/EGFP (tdTomato/eGFP) reporter mice (Strain #:007676; RRID: IMSR_JAX:007676; Jackson Laboratory) (IRcKO mice). Both male and female GlastCreERT2; IRf/f (IRcKO) and control (IRf/f) mice were 8-10 weeks old at the onset of Tamoxifen treatment, serving as the starting point for phenotype characterization
Wild animals	This study did not involve the use of wild animals
Reporting on sex	Male and females were used for this study. The sex of the animals was considered in the study design; it is specified in the figures the sex of the experimental groups.
Field-collected samples	This study did not involve the use of field collected samples
Ethics oversight	Care of animals was within institutional animal care committee guidelines. All procedures were reviewed and approved by the University of Santiago de Compostela Ethics Committee following the European Union normative for the use of experimental animals (Project ID 15012/2021/011).

Note that full information on the approval of the study protocol must also be provided in the manuscript.
